# Genomic Motifs as a Novel Indicator of the Relationship between Strains Isolated from the Epidemic of Porcine Epidemic Diarrhea in 2013-2014

**DOI:** 10.1371/journal.pone.0147994

**Published:** 2016-01-25

**Authors:** Takehisa Yamamoto, Tohru Suzuki, Seiichi Ohashi, Ayako Miyazaki, Toshiyuki Tsutsui

**Affiliations:** Virology and Epidemiology Research Division, National Institute of Animal Health, National Agriculture and Research Organization, Tsukuba, Ibaraki 305–0856, Japan; Sun Yat-sen University, CHINA

## Abstract

Porcine epidemic diarrhea virus (PEDV) is a positive-sense RNA virus that causes infectious gastroenteritis in pigs. Following a PED outbreak that occurred in China in 2010, the disease was identified for the first time in the United States in April 2013, and was reported in many other countries worldwide from 2013 to 2014. As a novel approach to elucidate the epidemiological relationship between PEDV strains, we explored their genome sequences to identify the motifs that were shared within related strains. Of PED outbreaks reported in many countries during 2013–2014, 119 PEDV strains in Japan, USA, Canada, Mexico, Germany, and Korea were selected and used in this study. We developed a motif mining program, which aimed to identify a specific region of the genome that was exclusively shared by a group of PEDV strains. Eight motifs were identified (M1–M8) and they were observed in 41, 9, 18, 6, 10, 14, 2, and 2 strains, respectively. Motifs M1–M6 were shared by strains from more than two countries, and seemed to originate from one PEDV strain, Indiana12.83/USA/2013, among the 119 strains studied. BLAST search for motifs M1–M6 revealed that M3–M5 were almost identical to the strain ZMDZY identified in 2011 in China, while M1 and M2 were similar to other Chinese strains isolated in 2011–2012. Consequently, the PED outbreaks in these six countries may be closely related, and multiple transmissions of PEDV strains between these countries may have occurred during 2013–2014. Although tools such as phylogenetic tree analysis with whole genome sequences are increasingly applied to reveal the connection between isolates, its interpretation is sometimes inconclusive. Application of motifs as a tool to examine the whole genome sequences of causative agents will be more objective and will be an explicit indicator of their relationship.

## Introduction

Porcine epidemic diarrhea virus (PEDV), a member of the Coronaviridae family, and genus Alphacoronavirus, is an enveloped, single-stranded, positive-sense RNA virus, which causes infectious gastroenteritis in pigs [1]. The virus has a ~28 kb genome containing a 5′ untranslated region (UTR), a 3′ UTR, and at least seven open reading frames (ORFs). The ORFs encode four structural proteins (spike (S), envelope (E), membrane (M), and nucleocapsid (N)), one hypothetical accessary protein (ORF3), and two polyproteins (1a and 1b), in the order of 5′UTR—ORF 1a/1b—S—ORF 3—E—M—N—3′UTR [2,3].

Since late 2010, PED outbreaks, which cause severe diarrhea and high mortality, have affected 80–100% of piglets in China [4–6]. Although a large proportion of the pig population had been vaccinated (using a vaccine derived from the CV777 strain), the protection was limited. Consequently, the outbreaks caused serious economic loss to the pig industry [4–7]. In the USA, the first case of PED emerged with very high mortality (90–95%) in suckling piglets in April 2013 [8]. Subsequently, the outbreak spread throughout North America, affecting more than 31 USA States as of July 2014 [9]. In the analysis of the genomes of the PEDV strains detected in the USA, most were grouped in the high mortality type similar to the Chinese PEDV strain (AH2012), and the remaining strains were grouped in the INDEL type, which have specific insertions and deletions in the S gene [9,10]. During 2013–2014, PEDV was obtained from farms showing high infectivity and/or mortality in suckling piglets in many countries worldwide such as South Korea [11], Japan [12], Taiwan [13], Canada [14], Mexico [15], Germany [16,17], and France [18]. The PEDV strains detected in these countries were grouped as US-like PEDV strains based on phylogenetic analysis using whole-genome sequences. The high similarity of whole-genome sequences among these PEDV strains, rather than the past strains detected in each country, led to the hypothesis that the origin of these virulent strains may be common [12,13].

Although a phylogenetic analysis was conducted for the PEDV strains discovered in the recent outbreaks, inconsistencies have arisen when results were compared between different regions of the genome or between results using partial and whole genome sequences [9,19–23]. Recombination has been suggested as a reason for these inconsistencies [9,19,22,24,25], as it is assumed to play important roles in escaping from vaccine protection and adapting to the host species [21,25,26]. For instance, if a specific region of the genome sequence was inserted in parental strains as a result of recombination, the region will be passed on to the daughter strains. The hypothetical existence of a partial genome sequence specific to the daughter strains incited us to explore partial genomic features shared by some PEDV strains as an indicator of the epidemiological association between PEDV strains detected in different locations.

## Materials and Methods

### Ethic statement

All fecal samples of Japanese isolates were submitted from Livestock Hygiene Service Centers in each prefecture to National Institute of Animal Health during disease control activities of local official veterinarians during 2013–2014 epidemic [12]. Therefore, all fecal samples had been collected passively from pigs with clinical signs such as diarrhea, and no aggressive operation had been conducted against pigs for sampling purpose. All samples were included in our previous study [12] under sample specific permissions from the local government which provided the relevant sample. Submission of fecal samples was conducted under the supervision of Ministry of Agriculture, Forestry and Fisheries. Sequences of non-Japanese isolates were downloaded from the GenBank database.

### Whole genome sequence of PEDV strains

From October 2013 to October 2014, intestinal samples from sows and/or piglets showing vomiting, anorexia, and watery diarrhea at 36 farms ware submitted from Livestock Hygiene Service Centers in 17 prefectures in Japan to National Institute of Animal Health (NIAH) during control activities against PEDV spread [12]. The virus isolation, RNA extraction, and sequencing were conducted in NIAH as described previously [12]. Whole genome sequences of PEDV detected in countries other than Japan were obtained from the GenBank database. To ensure the date of isolation of each PEDV strain was accurate, only the strains with sufficient information [9,10,14,17,27–31] were selected. As a result, 119 PEDV strains, including 36 Japanese and 83 foreign PEDV strains (76 from USA, 1 from Canada, 2 from Mexico, 1 from Germany, and 3 from Korea) were used in this study. The origins of and the date the pig illness was diagnosed at the farm as PED (for Japanese PEDV strain) or the date of detection (for foreign strains) are summarized in S1 Table. GenBank accession numbers of each PEDV strain are also included in S1 Table. For further analysis, we aligned all 119 PEDV sequences using the ClustalW method [32] in the MEGA6 program [33] with default parameter settings. The length of the aligned sequences was 27,746 nt. Phylogenetic analysis using the entire genome was performed by the MEGA6 program using the maximum-likelihood method with the general time reversible nucleotide substitution model. The confidence level for each branch was tested by the bootstrap method with 1,000 replicates.

### Determination of a sequence motif

A motif is generally defined as a nucleotide sequence that has some biological significance, such as a binding site on a regulatory protein. A variety of computational tools [34,35] have been developed for finding motifs on genome sequences. In this study, we defined a motif as a highly conserved region at the same position in the genome that was exclusively shared between several of the PEDV strains, assuming that a region of the genome replaced as a result of recombination will be passed on to the daughter strains. To find motifs based on our definition, we developed an original motif mining program using R software version 3.1.1 (R Core Team (2014), Vienna, Austria. http://www.R-project.org/) and “ape” package [36].

The process of identifying motifs is further detailed here. First, we removed identical sites among the 119 PEDV strains, and then defined the remaining 1,071 sites as single nucleotide polymorphism sites (SNPs). These sequences were then scanned using the defined window size (200 nt as a default value) starting from each SNP to find a motif and to determine the strains with the motif. For each selected window, every possible pattern of dividing the 119 strains into two groups was examined and if a statistically significant difference was found between these two groups, this window was determined to be a candidate motif region. The difference between these groups was obtained as a minimum value of differences between all possible pairs of strains from each group. The difference between strains was calculated as the total number of transitions, transversions, insertions, and deletions in SNPs in the region. The cut-off value for statistical significance was set as the upper 95% confidence limit of the Poisson distribution with the expected mean being equal to the mean of all prescribed pairwise differences in this window. For each candidate motif region, the strains in the minor group were the strains possessing the motif.

Since the candidate motifs were identified as genome regions and some of the windows analyzed contained multiple SNPs, continuous or adjacent candidate motifs were combined if they were shared with two or more strains that were identical in all relevant motifs. Finally, to reflect the combination process of motifs, the presence of each combined motif was tested for all 119 PEDV strains. When the difference between strains having the motif was less than 10% of the number of SNPs in the motif, the strain was determined to have the motif. Typically, the first strain to have a particular motif was determined as the reference strain for each motif. Starting and ending sites for each motif were identified with reference to the whole genome sequence of the referential PEDV strain, Colorado/USA/2013.

### BLAST search for the identified motifs

In order to explore the parental strain of the identified motifs, the nucleotide sequence within each motif of its reference strain was searched in GenBank by using BLAST (http://blast.ncbi.nlm.nih.gov) with its default parameters. Similarity of the motif between the reference strain and strains found by BLAST was calculated as a distance value as described previously.

## Results

### Overview of detected sequence motifs

**Fig 1:**
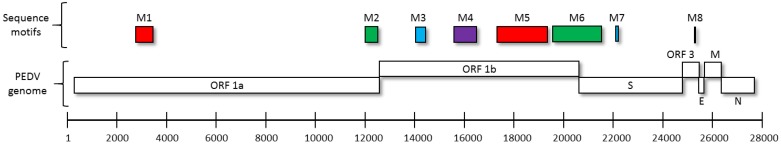
Location of the sequence motifs in the genome of PEDV. The numbers on the x-axis represent the position of the motifs in the PEDV genome as the number of nucleotides from the 5’UTR.

As a result of the use of the motif mining program over the genome sequences, eight motifs defined as M1–M8 were identified. These motifs are listed in Table 1, and a summary table for the presence of these motifs in the 119 PEDV strains is shown in S2 Table. The longest motif was M5 (2,032 nt, 79 SNPs), and the shortest motif was M8 (34 nt, 5 SNPs). Differences between the reference strain and the motif-positive and -negative strains was distinct. For example, the difference between the reference strain and the 14 strains having motif M6 was 0–7 (99.6–100.0% agreement), while that to the 105 strains without motif M6 was 149–153 (92.2–92.4% agreement). The position of the detected motifs was identified in the reference genome of the USA PEDV strain, Colorado/USA/2013 (Fig 1). All eight motifs were distributed along with the ORF 1a, 1b, S, and ORF3 genes. M6 was the only motif spanning different genes (i.e., from the 3′ end of the ORF 1b gene to the 5′ end of the S gene).

**Table 1:** Summary of sequence motifs identified within the genomes of 119 PEDV strains isolated from 2013–3014.

ID	Nucleotide position^a^		Length (nt)	SNPs^b^	Reference^c^	Strains^d^	Distance within motif						Agreement with reference strain					
							Positive strains			Negative strains			Positive strains			Negative strains		
	Start	End					Min		Max	Min		Max	Min		Max	Min		Max
M1	2,759	3,463	705	37	I	41	0	-	3	19	-	21	99.5	-	100	97.0	-	97.3
M2	12,016	12,541	526	27	I	9	0	-	1	5	-	8	99.8	-	100	98.5	-	99.1
M3	14,052	14,469	418	11	I	18	0	-	0	4	-	7	100	-	100	98.3	-	99.0
M4	15,594	16,503	910	37	I	6	0	-	3	16	-	19	99.7	-	100	97.9	-	98.2
M5	17,340	19,371	2,032	79	I	10	0	-	2	49	-	56	99.9	-	100	97.2	-	97.6
M6	19,569	21,530	1,968	203	I	14	0	-	7	149	-	153	99.6	-	100	92.2	-	92.4
M7	22,110	22,204	95	15	O	2	0	-	0	6	-	8	100	-	100	91.6	-	93.7
M8	25,282	25,315	34	5	K	2	0	-	0	5	-	5	100	-	100	85.3	-	85.3

^a^ Positions in Colorado/USA/2013;

^b^ Number of sites with one or more inconsistency within the 119 strains.;

^c^ The first isolated strain having the sequence motif. Abbreviations are for the following strains: I, Indiana12.83/USA/2013; O, Oklahoma35/USA/2013; K, KNU-1305/KOR/2013.

^d^ Number of strains having the motif.

### Motifs in the 119 PEDV strains

**Fig 2:**
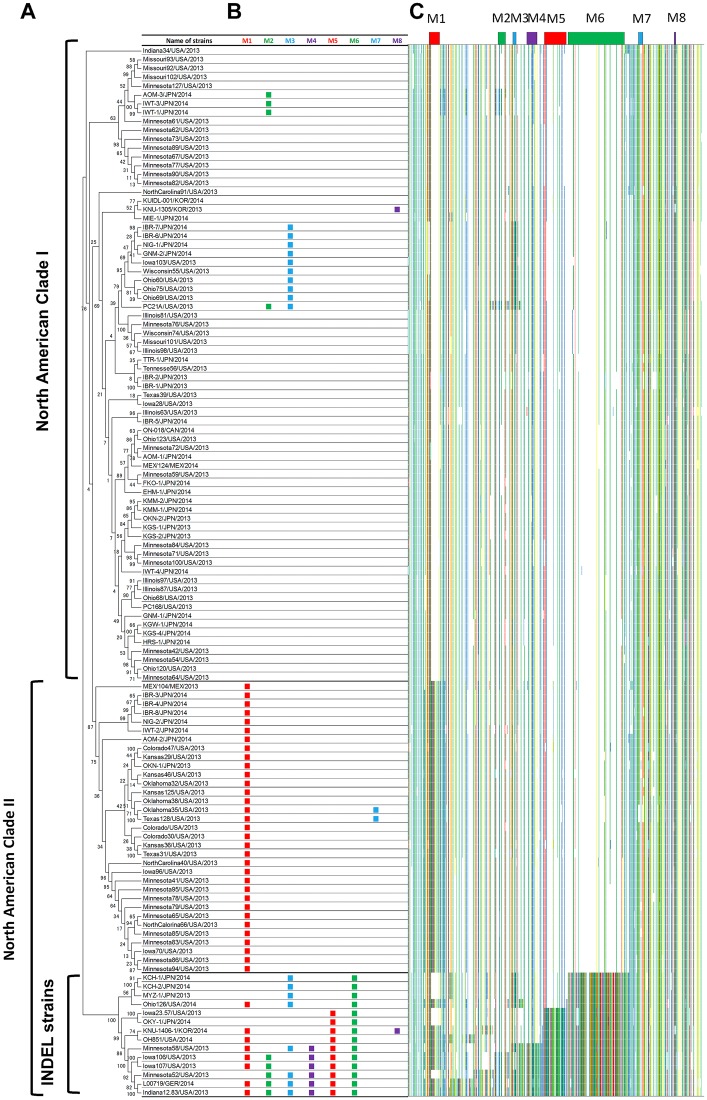
Phylogenic tree for 119 PEDV strains and identified motifs. (A) Phylogenetic tree based on the genomes of 119 PEDV strains isolated in 2013–2014. Phylogenetic analysis was performed using a maximum-likelihood method with general time reversible nucleotide substitution model and with a bootstrap test using 1000 replicates in the MEGA6 program. Notations on the very left side represent the clades shown by Vlasova et al. (9). (B) The presence of sequence motifs in each strain. (C) Color chart of nucleotides in the sites having inconsistencies within the 119 PEDV strains. Nucleotides in agreement with the sequence of Indiana12.83/USA/2013 are colored with respect to the type of nucleotide (a: red, t: blue, c: green, and g: yellow). To increase the discriminability of motifs, nucleotides in sites with only one inconsistent strain were not colored.

The phylogenetic tree of whole genomes from all 119 PEDV strains is shown with the annotation of clades reported by Vlasova et al. [9] in Fig 2A. In Fig 2B, the presence of motifs in each strain is shown by squares. The motif M1 appeared most frequently (41 strains) and motifs M7 and M8 appeared least frequently (2 strains). No motif was shared by an identical subset of PEDV strains. The motifs M1, M2, M3, and M8 were found in both the non-INDEL and INDEL strains, while M4, M5, and M6 exclusively appeared within INDEL strains. Among the motifs that were exclusively observed in the INDEL strains, motif M6 was present in all INDEL strains. Moreover, the number of strains that possessed a motif decreased in the order of M6, M5, and M4. The maximum number of motifs in one PEDV strain was six, and this was observed in two strains: Indiana12.83/USA/2013 (detected in the USA in 2013) and L00719/GER/2014 (detected in Germany in 2014). The motifs M7 and M8 were found in only two strains from USA and Korea, respectively. To validate the functionality of our motif mining program, the presence of these motifs was graphically compared with the actual nucleotide sequences within PEDV strains using the strain Indiana12.83/USA/2013 as a reference (Fig 2C). Regarding the six motifs found in Indiana12.83/USA/2013 (M1–M6), the presence of nucleotides that were identical to those found in the reference strain (densely colored area) at the position of each motif was clearly consistent with the presence of the corresponding motif. Conversely, with respect to motifs M7 and M8, which were not found in Indiana12.83/USA/2013, nucleotides that were inconsistent with the nucleotides present in the reference strain (sparsely colored area) were found at the position of these motifs.

### BLAST search for the identified motifs

Since the strain Indiana12.83/USA/2013 had six motifs (M1–M6), it was provisionally defined as the reference strain for the six motifs in the BLAST search. The motifs M3, M4, and M5 were almost identical to the corresponding sequence in ZMDZY (KC196276), which was the PEDV strain detected in 2011 in China [37]. The differences between the reference strain and the strain ZMDZY at these three motifs were between 0–3 nt (i.e., 99.8–100% identical). For the M6 motif, some global INDEL strains [9] such as virulent DR13 (JQ023161) detected in Korea in 1999 [38], CH/S (JN547228) detected in China in 1986 [37], and CV777 detected in Belgium in 1977 [39] were found nearest among the strains detected before 2013. The difference between these strains and the reference strain for M6 was 38–66 nt (i.e., 96.7–98.1% identical). The motif M1 was similar to the strain PEDV-1C (KM609203) detected in China in 2012 [20] with a 2 nt difference (i.e., 99.7% identical), and M2 was similar to the PEDV strains detected in China in 2011–2012 [21] such as GD-1 (JX647847), 7C (KM609204), CHGD-01 (JX261936), GD-A (JX112709), and ZJCZ4 (JX524137) with a 2 nt difference (i.e., 99.6% identical).

## Discussion

Elucidation of the relationship between strains of causative agents during a disease epidemic can help reveal transmission routes and allow for adequate control measures to be put in place to prevent further spread [40,41]. Phylogenetic tree analysis and whole-genome sequencing are the most recent and powerful tools for deciphering this relationship [40,41]. However, the interpretation of the results obtained from phylogenetic analyses can be controversial, especially when results were compared between different regions of the genome or between results using partial and whole genome sequences[9,19,22]. Thus, we utilized whole-genome sequencing to identify motifs that would reveal the genetic relationship between the 119 PEDV strains detected in PED outbreaks worldwide during 2013–2014. In doing this, we discovered eight sequence motifs. The agreement between the prevalence of these motifs and the sequence similarity between strains (Fig 2C) suggests that these motifs reflect actual genetic similarity among these PEDV strains. Considering the size of the motifs (34–2,032 nt) and the number of SNPs discontinuously included in each motif and identical to strains having the motif (smallest was 4 nt in motif M3), it is reasonable to suppose that the presence of these motifs was a result of an epidemiological connection between strains having the same motif and not the result of random genetic evolution in each distinct strain. Therefore, even if a motif is shared by strains in distant phylogenetic clusters, this analysis would suggest the presence of an epidemiological relationship between these strains. Moreover, the interpretation of the results from a phylogenetic tree analysis sometimes requires arbitrary aggregation of a complicated structure of roots into inclusive subgroups such as clades [9]. However, utilizing sequence motifs is a more objective and explicit indicator of the relationships between strains and will provide new insights into the route of disease transmission.

With regard to the features of motif-based analysis, its results were both consistent and inconsistent with the result of conventional phylogenic tree analysis. Among the 119 PEDV strains included in this study, 72 strains from the USA and 2 strains from Mexico were already analyzed by phylogenic tree analysis in a previous study [9]. An example of the consistency between the two methods can be observed with all eight strains grouped as a single clade named US INDEL strains by phylogenic analysis, which were also found to have the same motif M6 in this study (Fig 2B). Although the five US strains, Ohio59/USA/2013, Ohio75/USA/2013, Ohio60/USA/2013, Wisconsin55/USA/2013, and Iowa103/USA/2013, were grouped in the same sub-branch in North American clade I by phylogenic analysis, the difference between these five strains and the other strains in the same clade was not clear. However, as a result of the motif-based analysis, these strains were found to exclusively share motif M3, which was also found in some INDEL strains such as Minnesota58/USA/2013. This may suggest a possible epidemiological relationship between these strains although this was not obvious in the phylogenic tree analysis.

The motifs M1–M6 were shared by the PEDV strains from two or more countries and the most frequently observed motif (M1) was found in the PEDV strains from all six countries included in this study. Consequently, the PED outbreaks in these countries in 2013–2014 might be closely related, and may be a result of multiple transmissions of PEDVs between these countries and not a single invasion across their borders. This is similar to the results presented by Vlasova et al. [9] who suggested that there were multiple transmissions of PEDV from affected countries into USA by showing that several strains grouped into different clades were sourced from different countries. These results bring attention to the possibility of a non-accidental route of virus transmission between countries, such as commodities that are excluded from border control measures.

Interestingly, all six motifs (M1–M6) were found in the two strains: Indiana12.83/USA/2013 and L00719/GER/2014. Since Indiana12.83/USA/2013 was the first strain detected (in June 2013) among the 119 strains included in this study, this strain may be the source of all six motifs. There were strains that were detected in the USA earlier than Indiana12.83/USA/2013 (i.e., Iowa/16465/2013, KF452322 and USA/Indiana/17846/2013, KF452323), which occurred in April and May of 2013, respectively [8]. However, they were not genetically similar to Indiana12.83/USA/2013, but were similar to strains that had no motif in this study such as Iowa28/USA/2013. We conducted a BLAST search for the parental strain of the motifs M1–M6, and the strain ZMDZY detected in China in 2011 was assumed as the parental strain for motifs M3, M4, and M5 but not for M1, M2, and M6. The motifs M1 and M2 were thought to originate from the other Chinese strains detected in 2011–2012, such as PEDV-1C, GD-1, and PEDV-7C. The parental strain for M6 was not clear because no previous strain possessed a high identity with this motif. The inconsistency in the putative parent strains for each motif may suggest a complexity to the origin of these motifs found in the strain Indiana12.83/USA/2013.

Since the presence of motifs are the result of expression of certain unique genome sequence(s) in a subgroup of PEDV strains, their presence may influence viral characteristics of host strains. For instance, the S gene of PEDV, in which two motifs (partial M6 and M7) were found (Fig 1), is reported to regulate growth adaptation and pathogenicity [15,42,43], as well as induce neutralizing antibodies [15,42,44]. Likewise, motif M8 was found in the ORF 3 gene, which is reported to related to attenuation and virulence [42,45]. In addition to the application of motifs as epidemiological markers, elucidation of the relationship between motifs and viral features should be a target for future study.

Some things should be considered when attempting to utilize sequence motifs as an indicator of the epidemiological relationship between causative agents of infectious diseases. First, since uniqueness of the nucleotide sequence in a subgroup of examined strains is important in identifying a motif, the strains that are extremely different from the other strains should not be included in the examined strains. The reason for this is that such strains may increase the number of inconsistent sites, which may not contribute to the discrimination of a motif. For this purpose, the year of isolation for the target strains in this study was limited to a short period of time between 2013 and 2014. In addition, in the case where strains form a single focal epidemic such as an outbreak of highly transmissible exotic disease within an area, finding a motif might be difficult because the difference between strains would be small throughout all strains and finding a significant difference that separates the strains into subgroups would be difficult. Finally, estimation of a strain’s evolutionary history based on the profile of detected motifs is not plausible in this study as some of the identified motifs are in close proximity to each other on the PEDV genome, and thus the possibility of more than one motif being inserted or removed on a single occasion cannot be discounted. With this in mind, future studies will need to develop a reliable model to estimate the evolutionary history of these motifs from the difference in the pattern of the identified motifs.

## Conclusions

We identified eight sequence motifs from the genomes of PEDV strains detected in six countries during the 2013–2014 epidemic. Using the motifs as an indicator of an epidemiological relationship, we suggested that there may have been multiple transmissions of PEDV between these countries. While the recent advances in next generation sequencing have provided an opportunity to use whole-genome sequencing in epidemiological research, a technique to reveal relationships that are hidden in the information available in huge datasets are still being developed [40,41]. The analysis of sequence motifs shown here is a novel tool to evaluate the relationship between strains of causative agents using whole genome sequence data.

## Supporting Information

S1 File R codes for finding motifs. The zip file contains two R code files. “SNP scan” code is for finding candidate motifs from a set of viral sequences. The “Motif scan” code is for identifying motifs found by “Motif scan” code in each sequence. Sequences should be aligned and trimmed such that all sequences have the same length and should be saved in the fasta format (.fas file). All candidate motifs should be aggregated when adjacent motifs were shared by the same subgroup of sequences and written in the “Motif address_xxx.csv” file before running “Motif scan” code.
(ZIP)

S1 Table List of PEDV strains and motifs. Detail of 119 PEDV strains examined in this study with motifs in each strain. All strains listed in the order of identified country and date of identification. GenBank accession numbers are also included.
(XLSX)

S2 Table Detail of nucleotides in the motifs. Excel workbook with spreadsheets for the detail of nucleotides in SNPs contained in each motif. Location of each SNP is shown as the number of nucleotides from the 5’UTR of PEDV strain Colorado/USA/2013. Nucleotides identical to the reference strain for each motif at each site is shown as dots. Notations for nucleotides follows IUPAC rule; a: adenine, g: guanine, c: cytosine, t: thymine, n: a, g, c or t, k: g or t, m: a or c, y: c or t.
(XLSX)
